# A price comparison of recently launched proprietary pharmaceuticals in the UK and the US

**DOI:** 10.3402/jmahp.v4.32754

**Published:** 2016-09-12

**Authors:** Jesper Jørgensen, Panos Kefalas

**Affiliations:** Cell and Gene Therapy Catapult, Guys Hospital, London, UK

**Keywords:** pharmaceuticals, pricing, orphan, ultra-orphan, high-cost, highly specialised therapies, cell therapy, gene therapy

## Abstract

**Objective:**

To explore the relationship between prices charged by manufacturers of proprietary pharmaceuticals in the US and in the UK in recent years (2013–2016), expressed as a multiplier, and to detail to what extent this relationship differs for high-cost therapies used in smaller patient populations, as compared to lower-cost drugs.

**Methodology:**

Therapies assessed by the Scottish Medicines Consortium (SMC) in the UK between 1 January 2013 and 1 June 2016 were identified; only in-patent therapies were included in the analysis (to avoid the impact of price erosion post patent expiry); results were grouped according to annual cost per patient (whether considered high-cost, i.e., >£2,500 per patient per year, or not) and the size of the UK target population [whether considered orphan (<32,000 patients per year), ultra-orphan (<1,000 patients per year), or not]. Publicly listed prices were obtained in the US and UK and were adjusted where necessary to estimate the prices charged by manufacturers in the respective countries. The difference in price (per unit of the same strength and formulation) was calculated as a multiplier between the US and UK prices for each of the therapies identified.

**Results:**

Based on the methodological approach described, 88 therapies were identified and included in the analysis. The multiplier between the US and UK prices was 3.64 for therapies with an estimated annual cost <£2,500; this was reduced to 1.90 for higher-cost therapies. A downward trend was also evident in the subgroup analysis of the higher-cost therapies; as the estimated target patient populations reduced from >32,000 down to <1,000, the US/UK price multipliers reduced from 2.13 for the former to 1.48 for the latter.

**Conclusion:**

Although pharmaceutical prices have been found to be on average substantially higher in the US compared to the UK, our findings suggest that this price discrepancy is smaller for higher-cost therapies targeting small patient populations. Manufacturers of high-cost products should therefore factor this in when formulating pricing strategies because the potential for higher pricing in the US seems greater for primary care products targeting large patient populations.

Drug prices vary greatly in different countries, and drug prices in the US are widely reported to be higher than in other countries (1–6). However, the price charged by manufacturers is usually lower than the cost to hospitals or patients. Manufacturers typically sell their products to wholesalers for what is referred to as the ‘ex-factory price’ (6), ‘ex-manufacturer price’ (7), or ‘wholesale acquisition cost (WAC)’ (8); subsequently, wholesalers apply a markup before selling these products to pharmacies who also apply a markup or pharmacy margin [in many countries value-added tax (VAT) also applies], and the sum of all these components constitutes the retail price. The magnitude of the wholesaler and pharmacy margins also differs among countries. In the US, the wholesaler and pharmacy margins account for about 3 and 22% of retail price, respectively (7). In the UK, the wholesale margin is reported to be no higher than 12.5%, and the pharmacy margin to be around 2% (9). This means that, although retail prices are substantially higher in the US than in the UK, the difference in the prices actually charged by manufacturers in the two countries is smaller.

The UK Department of Health (DoH) published an analysis of international medicine price comparisons. Their analysis found that the prices charged by manufacturers for primary care products in the US were 2.81 times higher than in the UK in 2010 (6) (the 2.81 multiplier was calculated using US and UK product prices weighted by their share of England community prescribing, at an exchange rate of 1.58 US dollars per GBP). Furthermore, the Association of the British Pharmaceutical Industry (ABPI) estimated a multiplier of 3.31 for 2011 based on the same data (10). Although the DoH and ABPI price comparisons are useful in establishing the US/UK price difference for larger-volume, primary care products, they fall short of identifying how this relationship might differ for higher-cost therapies used in smaller patient populations.

The objective of this analysis is to explore the relationship between prices charged by manufacturers in the US and in the UK in recent years (2013–2016), expressed as a multiplication factor, and to detail to what extent this relationship differs for higher-cost therapies used in smaller patient populations, as compared to lower-cost drugs. This information is particularly useful for manufacturers of therapies with high manufacturing costs (e.g., cell and gene therapies), which are dependent on justifying a high price in order to be commercially viable. Such therapies are usually being developed to target smaller patient populations with a high unmet need because the potential for improvement in patient outcomes is greater (which helps justify a higher price), and lower volumes of patients help reduce payers’ budget impact concerns and thus the risk of subsequent reimbursement restrictions (11).

## Methods

We identified the therapies assessed in the UK between 1 January 2013 and 1 June 2016 in the Advice Directory of the Scottish Medicines Consortium (SMC) (12). The SMC was chosen instead of the National Institute for Health and Care Excellence (NICE) because the former aims to assess every new licensed medicine launched in the UK within approximately 18 weeks (13), while the latter does not routinely assess all new market entrants (specific elimination, selection, and prioritisation criteria apply) (14). We limited the search to include only treatments classified as ‘accepted for use’ or ‘accepted for restricted use’; therapies ‘not recommended for use’, ‘withdrawn’, or ‘superseded’ were excluded.

Each therapy was categorised according to the following parameters:

- British National Formulary (BNF) category and indication under assessment as detailed by the SMC
- Patent protection status
- Size of target patient population (as defined in the manufacturer's submission; typically providing estimates for year one and five post-launch, of which we used the higher of the two)
- Estimated gross annual cost per patient (as defined in the manufacturer's submission; corresponding to the same year post-launch as noted in the previous parameter)

We then excluded therapies according to the criteria detailed in Table 1.

**Table 1:** Number of therapies identified in the original search and reasons for exclusions

		Exclusion criteria and number of therapies excluded			
			
BNF category	Original search (*N*)	Duplicates	Off patent	Patient numbers not stated^a^	Total (*N*) included in pricing analysis
Skin	8		5		3
Immunological products and vaccines	0				0
Eye	17	4	4	5	4
Ear, nose, and oropharynx	1		1		0
Respiratory system	14	1	3	2	8
Cardiovascular system	14	3	3		8
Musculoskeletal and joint diseases	10	3	2	1	4
Gastrointestinal system	10	5	2		3
Obstetrics, gynaecology, and urinary-tract disorders	9	1	6	1	1
Endocrine system	33	7	10	6	10
Central nervous system	16	4	6	2	4
Infections	36	8	4	11	13
Nutrition and blood	9		6		3
Malignant disease and immunosuppression	58	13	8	1	36
Total (*N*)	235	49	60	29^b^	97

a Most of which were abbreviated submissions with limited economic data.

b One of which was a therapy in an orphan indication [entecavir (Baraclude)], in paediatric chronic hepatitis B; the remaining 28 were in more prolific indications.

Subsequently, we gathered price information from the BNF on the Medicines Complete website (15). The prices listed in the BNF provide basic NHS prices that ‘do not take into account VAT, professional fees, and other overheads’ (16), meaning they provide a reasonable estimate of the prices paid to manufacturers. US prices were obtained from two sources: 1) the lowest price listed by a named pharmacy on the US retail pharmacy price comparison site GoodRx.com (17), adjusted for a pharmacy margin assumed to be 25% (7); and, in cases where prices were not available on GoodRx.com (typically for hospital-only products), 2) the WAC price as listed in the Red Book Online (18). Therapies for which prices were not available in the UK and/or the US were excluded from the analysis.

US prices were converted to British pounds (GBP) using the average exchange rate for 2016 (1.434 US dollars per GBP) obtained in May (19). We calculated unit prices in the US and UK (i.e., price per same strength pill, vial, or injection, etc.) for each therapy to allow comparisons in cases where pack sizes differ between the two geographies. For therapies where several strengths were available, we chose the dose that most closely matched the recommended average dose for adults, as per the BNF.

We grouped therapies according to (1) whether they can be considered high cost in the UK (i.e., annual cost per patient >£2,500)^1^ (20) and (2) whether their estimated UK target populations can be considered orphan (1,000–32,000)^2^ (21), ultra-orphan (<1,000)^3^ (22), or neither (>32,000). Estimates for annual UK costs per patient were calculated using figures from the SMC assessments for budget impact (i.e., the estimated number of patients) and gross budget impact according to projected market shares (as provided in the manufacturers’ submission). Estimates of the number of patients in the UK were calculated using the Scotland-specific figures from the SMC assessments multiplied by 12.^4^

Finally, we calculated the multiplication factor for each of the therapies (by dividing the US unit price by the UK unit price) as an expression of the price difference between the two countries. Confidence intervals (CIs) for the results in the different groupings (all therapies, therapies with annual cost per patient <£2,500, and >£2,500 including subgroups according to the estimated UK target population as detailed earlier) were calculated using the *t*-test.

## Results

The original search provided 235 results, from which 138 were excluded as detailed in Table 1 (based on the criteria listed earlier). Price information was sought for the remaining 97 therapies, and a further nine therapies were excluded at this point because their prices were not available in the UK and/or US. The US/UK multiplication factor was calculated for each of the remaining 88 therapies; furthermore, mean and median multipliers were calculated for the groupings described earlier (according to estimated UK annual per patient cost and patient numbers) as displayed in Table 2.

**Table 2:** Average US/UK price multipliers (and confidence intervals) according to annual average costs per patient and size of target patient population in the UK

Therapy groupings		*N*	Median US/UK multiplier	Mean US/UK multiplier	Confidence interval (CI) lower bound	CI upper bound	Average annual cost per patient (UK)
All		88	2.00	2.51	0.66	4.35	£15,923
Annual UK cost per patient <£2,500		30	3.62	3.64	1.97	5.32	£664
High-cost therapies (annual UK cost per	All with an annual UK cost per patient >£2,500	58	1.20	1.90	0.60	3.20	£23,815
patient >£2,500)	>32,000 UK patients per year	7	1.98	2.13	1.20	3.06	£8,877
	1,000–32,000 UK patients per year	39	1.81	1.99	0.56	3.42	£24,397
	<1,000 UK patients per year	12	1.50	1.48	0.66	2.29	£30,637

Where prices were available from both US sources, the retail prices listed on GoodRx.com were only 1.2% higher on average than the WAC prices listed on the Red Book Online. This indicates that the WAC prices as listed on the Red Book Online do not reflect the actual price paid to the manufacturer; we therefore adjusted these WAC prices for the average difference between the GoodRx.com price (minus the assumed margin of 25%) and the listed WAC price – a discount of 15.4%.

Our results showed that the difference (multiplier) between the US and UK prices is greater for lower-cost therapies, that this difference is reduced for higher-cost therapies, and reduced further in the orphan and ultra-orphan subcategories. This is illustrated by a mean multiplier of 1.90 (median of 1.20) for high-cost treatments as a whole, compared to a mean of 3.64 (median of 3.62) for the lower-cost therapies (which is in the same region as the 3.31 result found by ABPI in 2010^5^ (10). This trend is also evident in the subgroup analysis of the higher-cost therapies; as the estimated patient populations become smaller (i.e., from >32,000 down to <1,000), the US/UK price multipliers reduce, and the average annual costs per patient increase substantially.

Therapies categorised by the BNF as *malignant diseases* (e.g., oncology and leukaemias) and *immunosuppression* accounted for 34 of the 88 therapies identified, and this category was overrepresented among treatments with an estimated annual cost of >£2,500 (accounting for 32 of the 58 therapies in this group). Furthermore, nearly all (31) of these can be categorised as orphan and ultra-orphan (21/39 for orphan therapies and 10/12 for ultra-orphans).

**Fig. 1:**
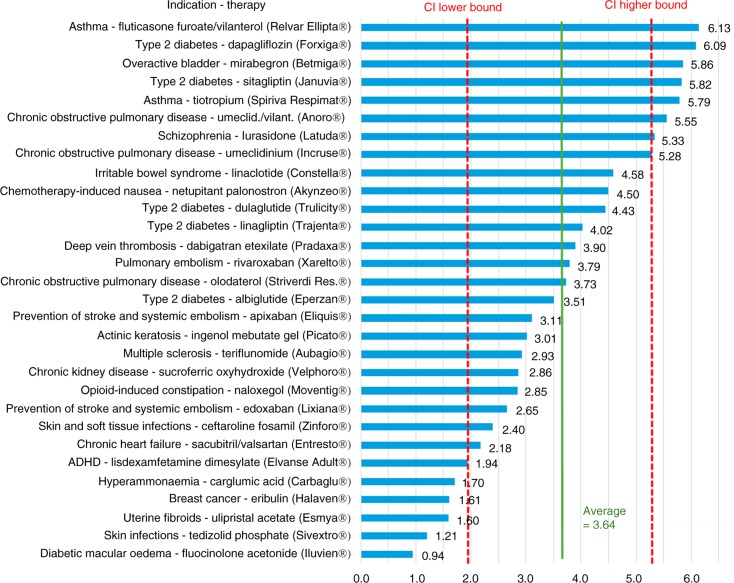
US/UK price multipliers for therapies with an average annual cost per patient <£2,500 in the UK.

**Fig. 2:**
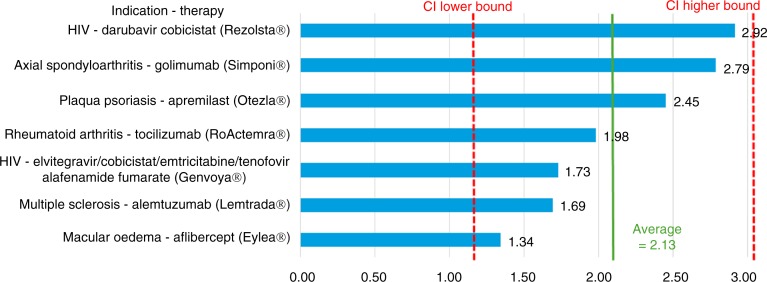
US/UK price multipliers for therapies with an average annual cost per patient >£2,500 in populations of >32,000 patients per year in the UK.

**Fig. 3:**
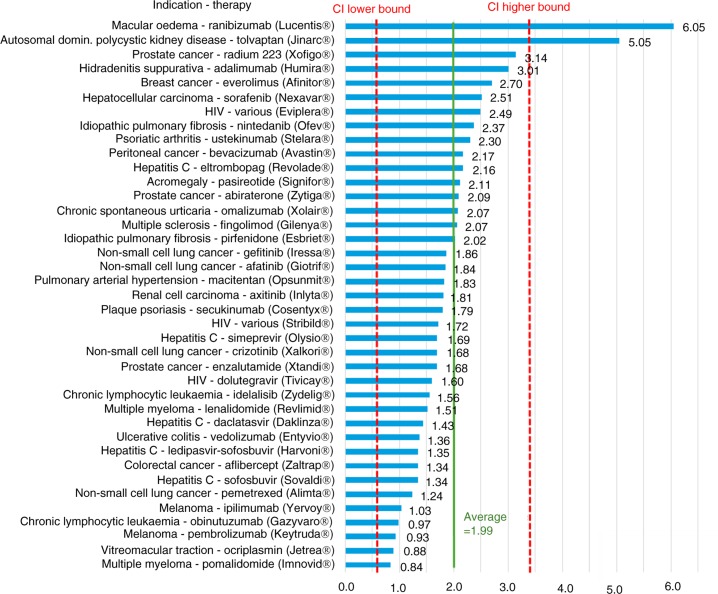
US/UK price multipliers for therapies with an average annual cost per patient >£2,500 in orphan-sized populations (1,000–32,000 patients per year) in the UK.

**Fig. 4:**
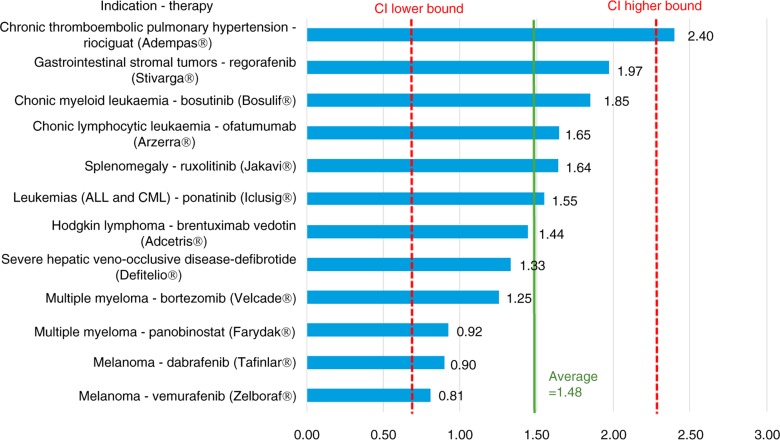
US/UK price multipliers for therapies with an average annual cost per patient >£2,500 in ultra-orphan-sized populations (<1,000 patients per year) in the UK.

Figure 1 provides detailed results for the lower-cost therapies (with an estimated annual cost per patient <£2,500), with a mean multiplier of 3.64 (with a 95% CI ranging from 1.97–5.32). Figures (2–4) provide similarly detailed results for the higher-cost therapies; Fig. 2 illustrates the results for the subgroup with an estimated target population of >32,000, with a mean of 2.13 (CI ranging from 1.20–3.06); Fig. 3 shows the results for the subgroup with an estimated target population of 1,000–32,000, with a mean of 1.99 (CI ranging from 0.56–3.42); and, finally, Fig. 4 shows the results for the subgroup with an estimated target population of <1,000, with a mean of 1.48 (CI ranging from 0.66–2.29).

## Discussion

Higher drug prices in the US compared to those in the UK have been widely reported (1–6); however, there are two factors that need to be considered. First, higher retail prices in the US do not necessarily reflect proportionally higher manufacturer selling prices because the wholesaler and pharmacy margins are higher in the US compared to the UK. Second, as this analysis demonstrates, the difference in prices decreases as we move from low-cost to high-cost therapies and from large to small patient populations. A key strength of our analysis is that it provides results specifically for products targeting small patient populations and, therefore, goes further in informing what the relationship between US and UK prices is for this subgroup of highly specialised therapies.

It is important to highlight that, given the overlapping CIs across the different therapy groupings analysed here, the results presented should only be interpreted as an overall trend.

It is also worth noting that our findings for the lower-cost therapies (i.e., mean multiplier of 3.64) are in the same region as the previously reported multipliers by the DoH and ABPI (i.e., 2.81 and 3.31, respectively; observed discrepancies are in part a reflection of differences in the exchange rates applied). When we apply to our analysis the exchange rate used in the DoH analysis^6^ (i.e., USD to GBP and an exchange rate of 1.58), we find that the multiplier for the treatments with an estimated annual cost per patient of <£2,500 matches the 3.31 estimated by the ABPI for 2011.

The exchange rate of 1.434 (GBP to USD) applicable to the time period during which this analysis was undertaken was reduced after the EU referendum (held in the UK on 23 June 2016); however, fluctuations in exchange rates do not alter the trend identified in our subgroup analysis (i.e., that US/UK price differences are reduced as the annual per patient costs increase and the size of the target populations decreases), and this remains a key strength of our analysis.

Furthermore, the DoH analysis, upon which the ABPI based its 2011 estimates, uses a broad average of prices for the top 250 branded primary care medicines in the UK and pooled data from therapies launched around 2010 as well as for products that had been on the market for longer. A second strength of our analysis is that it focuses solely on newly assessed products within a three-and-a-half-year time frame (2013–2016) and therefore provides a more contemporary perspective on the relationship between pharmaceutical prices in the UK and US. Also because we apply a tightly defined time frame in the analysis, the impact of price erosion over time is reduced.

Finally, it should be noted that the price estimates applied in our analysis may differ from the actual manufacturer selling prices because confidential discounts are often operational in both the UK and the US (1–9). However, due to the confidentiality of the discounts in the UK and the US, it is not possible to draw any conclusions regarding the net effect on the multipliers identified here.

## Conclusion

Although pharmaceutical prices have been found to be on average substantially higher in the US than in the UK, our findings indicate that, for higher-cost therapies that target small patient populations, this price discrepancy is smaller. Manufacturers of high-cost products should therefore factor this in when formulating pricing strategies and be cognizant that the differences in prices charged across the Atlantic may be limited compared to that of primary care products targeting large patient populations.

## Footnotes

1 NHS England's guideline when considering drugs for inclusion in the high-cost drug exclusion list states that ‘there is, or is expected to be, more than £1.5 million spent or 600 cases in England per annum’; £1.5 million divided by 600 is £2,500.

2 Five affected persons per 10,000, assuming a UK population of 64 million, equals 32,000.

3 NICE uses the ultra-orphan term for conditions occurring in less than 1,000 people in the UK.

4 The population of the UK is approximately 12 times that of Scotland.

5 However, the exchange rate applied in the DoH analysis (upon which ABPI based their 2011 results) reflected the average exchange rate of the fourth quarter of 2010, which was significantly higher (1.58) than the more recent exchange rate used in our analysis (1.434).

6 Which formed the basis for the ABPI analysis.
